# Direct Care Workers Retention and Recruitment Trends in Ohio Residential Care Facilities Pre-Post COVID-19

**DOI:** 10.1093/geroni/igaf122.3194

**Published:** 2025-12-31

**Authors:** Oksana Dikhtyar, Ian Matthew Nelson

**Affiliations:** Miami University, Oxford, Ohio, United States; Miami University, Oxford, Ohio, United States

## Abstract

Retention and recruitment of direct care workers (DCWs) in nursing homes and residential care facilities (RCFs) has been a challenge even before the COVID-19 pandemic, but became even more pronounced during and after. While there are various public data sources about nursing homes, the information about RCFs, most of which are assisted living facilities, is not collected. In Ohio, the Biennial Survey of Long-Term Care Facilities mandated by the state provides comprehensive data on RCFs. Using three waves of the Ohio Biennial Survey of Long-Term Care Facilities, we examined changes in retention and turnover of DCWs, facilities’ perceptions of the seriousness of the problem, recruitment approaches, and workplace environment and financial strategies for retention prior to, during, and post COVID-19 pandemic. The average retention rate of DCWs has increased from 64.7% in 2017 to 68.5% in 2023, but the turnover rate stayed the same at 78.3%. Four in ten facilities ranked DCW retention and recruitment as serious problem across all three waves. However, the proportion of facilities that had an insufficient number of qualified applicants fell by 10% during each wave and was 41.4% post COVID. There have been across-the-board increases in the proportion of facilities that implement a variety of workplace environment and financial strategies to retain workers. In terms of recruitment, almost twice as many facilities provided same day pay in 2023 compared to 2021 and 40% stopped requiring drug testing post COVID compared to 17.9% during COVID. Implications for policy and practice are discussed.

